# Autistic Children Are More Responsive to Tactile Sensory Stimulus

**Published:** 2018

**Authors:** Asmika ASMIKA, Lirista Dyah Ayu OKTAFIANI, Kusworini KUSWORINI, Hidayat SUJUTI, Sri ANDARINI

**Affiliations:** 1 Laboratory of Public Health Sciences, Faculty of Medicine, Brawijaya University, Jalan Veteran Malang 65145, East Java, Indonesia; 2 Graduate Program of Biomedical Sciences, Faculty of Medicine, Brawijaya University, Jalan Veteran Malang 65145, East Java, Indonesia; 3 Laboratory of Clinical Pathology, Faculty of Medicine, Brawijaya University, Jalan Veteran Malang 65145, East Java, Indonesia; 4 Laboratory of Biochemistry-Biomolecular, Faculty of Medicine, Brawijaya University, Jalan Veteran Malang 65145, East Java, Indonesia

**Keywords:** Autistic Children, Tactile Sensory, Short Sensory Profile

## Abstract

**Objective:**

This research was an experimental study that was aimed to detect differences response of tactile sensory stimulus between normal children and children with sensory brain development disorders such as Autism Spectrum Disorder (ASD).

**Materials & Methods:**

A total of 134 children, in two groups including 67 healthy children (control) and 67 children with autism were studied. Tactile sensory stimulus responses in children were tested directly using a Reflex Hammer. In addition, tactile sensory sensitivity was also assessed via questionnaire Short Sensory Profile (SSP) filled out by the child's parents. All response data were analyzed using Fisher's Exact Test; questionnaire data was analyzed with the Mann-Whitney U Test.

**Results:**

Autistic children were more sensitive to palpation and pain than children who were not autistic. Furthermore, the value of SSP was also significantly higher (*P*<0.05) in autistic children, which means that they always responded to all categories in the SSP questionnaire than children who are not autistic.

**Conclusion:**

Autistic children are more sensitive to tactile sensory stimulus and all categories of SSP than children who are not autistic.

## Introduction

Sensory organs are peripheral components of the somatosensory system whose function is to record physical and chemical changes in the external and internal environment of the body and turn them into electrical impulses that are processed by the nervous system (1). One of the largest sensory systems is located on the skin, which delivers information on stimuli such as touch, vibration, pressure, pain, and temperature. This sensory aspect reception pattern has occurred since the age of children to adult (2).

In children, this neurological system helps the process of categorizing and developing responses to information obtained from the environment. Each child has a different ability to process and to respond to information or stimuli from the environment (3). Normally, children who receive stimulation on the skin or body surface process it in the brain and then generate appropriate responses to the sensation or stimulus received (4). However, some children with sensory processing disorder find it difficult to interpret and to respond appropriately to the stimulus received (5).

Generally, children with autism syndrome have problems associated with neural development in the brain such that the processes of sensory integration in the brain are distracted (6). The Tactile Sensory system is a sensory system set up by tactile response and pain receptors in the skin (7); the Short Sensory Profile (SSP) is a method of measuring sensory system response using a questionnaire, usually used to determine the contribution of the sensory aspects of children in everyday life and can be used to explain differences in tactile sensitivity (8, 9).

We aimed to show differences in sensory integration in children with sensory brain development disorder (Autism Spectrum Disorder), especially in tactile sensory sensitivity and SSP values.

## Materials & Methods

Research Subjects 

This study used random sampling to get the respondents. Respondents were normal children who were in elementary school and autistic children at the Center for Autism Services, Extraordinary Schools and Inclusion Elementary School in Malang, Indonesia aged 6–13 year. This study was conducted in July-August 2016. Respondents totaled 134 children: 67 were normal children and 67 autistic children. Gender and demographic characteristics was not necessary for respondent selection as long as all respondents lived with their parents. 

This study received approval from the Research Ethics Committee of the Faculty of Medicine, Brawijaya University, Indonesia No.502/EC/KEPK/09/2015. 

**Tactile Sensory Measurement**

Tactile sensory sensitivity was measured using a Reflex Hammer due to it was the most popular tool in Indonesia and could provide reliable data. The testing procedure was applied along with SSP questionnaires (Supp.data1). The Touch Assessment Test (Reflex Hammer) touches and scratches the skin of the arm of the blindfolded respondents for 1.5 seconds at a random location. Then the respondents were asked to tell where on their arm they felt the stimulus touches. Reflex Hammers were used to view the tactile sensitivity to touch and pain response.

SSP questionnaires were given and filled by caregivers, consisted of 38 questions designed to uncover the sensory experiences in the children’s daily lives, using a tactile scale score. (Tomchek and Dunn, 2007). 

**Statistical Analysis**

The data obtained were tabulated and analyzed. The data’s sample characteristics, in general, were analyzed with descriptive statistics. The differences in tactile sensory response (tactile and pain response) between both groups were analyzed using Fisher's Exact Test, and in the SSP using the Mann-Whitney U Test. The entire analysis was using the statistical program SPSS for Windows version 17.0 (Chicago, IL, UA).

## Results

**General Characteristics of Respondents**

76.1% of the respondents were male while 29.9% of the normal children’s parents did not graduate from elementary school. It was contrasted with the autistic children’s parents who had graduated from junior high school, senior high school and vocational school to master degree. Almost all respondent’s fathers have job (Table 1). 

**The difference of the Tactile Sensory Response between Normal and Autistic Groups**

The differences of the tactile sensory response of touch and pain between both groups were analyzed using Fisher's Exact Test. Statistical test results showed significant differences in all categories of tactile sensory responses in both groups (*P* = 0.001). Children with autism were very sensitive to the touch and pain responses (Table 2).

**The Differences of Short Sensory Profile (SSP) Values between Normal and Autistic Groups**

SSP values were analyzed using Mann-Whitney U Test. Statistical test results showed significant differences of SSP values in both groups (*P* = 0.001) (Table 3). Autistic children were always responding to all category of SSP questionnaire (Table 4).

## Discussion

Early detection for autism condition is necessary for further treatment. This study provided basic knowledge to understand autism condition. In individuals with good sensory integration, the brain has the ability to organize and process sensory input and use that input to respond appropriately to outside stimuli. However, some children with sensory processing disorder find it difficult to interpret and to respond appropriately to the stimulus received (5). Children with autism syndrome have problems associated with neural development in the brain so that the processes of sensory integration in the brain are distracted. This disturbance in the system impacts the recording and interpretation of sensory inputs, resulting in problems in learning, development, and/or behavior (10).

The results showed that the group of autistic children were more sensitive to touch and pain response than the group of normal children. Moreover, the results of the questionnaire SSP completed and reported by parents about their children in this study also showed that the group of autistic children always responded to all categories in the questionnaire SSP: tactile, taste, move, sensation of seeking, auditory, weakness, and visual aspect compared to the group of normal children. Younger children are more likely to exhibit sensory hyperresponsiveness than older children. Children with ASD were reported that they are over-responsiveness (11). The results of that study indicated that ASD children were significantly more sensitive/over-responsive compared to controls. There was also a correlation between over-responsiveness with the tactile stimuli from parent reports and a lack of socializing (12). Besides, children with autism could be clearly seen to have sensory processing disorders. They tend to be more responsive to stimuli received compared to normal children.

**Table 1:** General Characteristic of Respondents

**Category**	**Group**			
	**Normal (n=67)**		**Autistic (n=67)**	
	**n**	**%**	**n**	**%**
**Ages (** **yr** **)**				
6-8	1	1.5	22	32.8
9-11	42	62.7	32	47.8
≥12	24	35.8	13	19.4
**Gender**				
Male	29	43.3	51	76.1
Female	38	56.7	16	23.9
**Fathers Recent Education**				
Uneducated	20	29.9	1	1.5
Elementary School Graduate	12	17.9	5	7.5
Junior High School Graduate	14	20.9	12	17.9
Senior High School Graduate	15	22.3	31	46.3
Degree Graduate	6	9.0	18	26.8
**Mothers Recent Education**				
Uneducated	20	29.9	1	1.5
Elementary School Graduate	14	20.9	8	11.9
Junior High School Graduate	9	13.4	14	20.9
Senior High School Graduate	23	34.3	23	34.3
Degree Graduate	1	1.5	21	21.4
**Father's Occupation**				
Have no job	17	25.4	2	3.0
Have job	50	74.6	65	97.0
**Mothers Occupation**				
Unemployed	51	76.2	41	61.2
Work	16	23.8	26	38.8

Note: n: real number of participant; %: percentage ratio in the group

**Table 2:** The difference of Tactile Sensory Response Touch and Pain Category

Tactile Sensory Response		Group				Fisher's Exact Test (p)
		Normal		Autistic		
		n	%	n	%	
**Touch**	None	66	98.5	8	11.9	0,001
	Sensitive	1	1.5	59	88.1	
**Pain**	None	61	91.0	3	4.5	0,001
	Sensitive	6	9.0	64	95.5	

Note: n: real number of participant; %: percentage ratio in the group

* Within a row, values with different superscripts are significantly different, *P* < 0.05. n =134

**Table 3:** The Differences of Short Sensory Profile (SSP) Value

SSP Category	Group		Mann-Whitney Test (p)
	Normal (Mean Rank)	Autistic (Mean Rank)	
Tactile	47.15	87.85	0.001
Taste	52.48	82.52	0.001
Move	57.48	77.54	0.001
Sensastion of Seeking	39.11	95.89	0.001
Auditory	41.43	93.57	0.001
Weakness	51.76	83.24	0.001
Visual	48.96	86.04	0.001

**Table 4:** Differences in the Autism and Control group's Short Sensory Profile (SSP) based on parent reports

Category	Autism (Mean-rank)	Control (Mean-rank)	*P*
**Touch Sensitivity**			
• Looks unhappy when asked to tidy up (eg wearing clothes and combing hair)	62,14	73,95	0,010
• Prefer long-sleeved clothes when hot; Short sleeves when cold	62,59	73,49	0,002
• Avoid barefoot roads, especially in grass or sand	58,66	77,48	0,0001
• React emotionally or aggressively to touch	54,74	81,46	0,0001
• Likes to avoid splashing water	67,52	68,49	0,662
• Have difficulty standing near other people	64,56	71,49	0,017
• Rub or scratch the other person touches	66,54	69,49	0,254
**Sensitivity of Flavor**			
• Avoiding certain flavors of food or the smell of food that is usually part of a child's diet	53,74	82,47	0,0001
• Just want to eat foods with a certain flavor	61,64	74,44	0,008
• Restrict eating textured foods (eg solid/flaccid, rough/soft foods or temperature (hot/cold))	60,17	75,95	0,001
• Picky eater especially related to the texture of the food	60,65	75,46	0,002
**Motion Sensitivity **			
• Become more anxious or depressed when the feet do not step on the ground	66,54	69,49	0,254
• Fear of falling when in height	62,65	73,43	0,028
• Do not like the head in upside down position	60,65	75,46	0,001
**Sensation of seeking**			
• Love foreign sounds or search for sound sources	51,31	84,94	0,0001
• Pay attention to all movements and disrupt routine activities	51,27	84,98	0,0001
• Too excited during mobile activities	50,88	85,37	0,0001
• Touching people and objects / likes to touch certain parts of an object and person	45,35	90,99	0,0001
• The face with no expression	56,68	79,49	0,0001
• Moving from one activity to another	49,81	86,46	0,0001
• Letting her clothes wrap around or tight on the body	62,11	73,98	0,003
**Hearing Information**			
• Interrupted or having difficulty functioning in noisy environments	66,57	69,46	0,431
• Does not seem to hear or empathy with what people say	48,80	87,49	0,0001
• Can not work in noise situations	56,74	79,43	0,0001
• Having difficulty completing tasks when radio is turned on	56,70	79,47	0,0001
• Does not respond when his/her name is called	58,16	77,99	0,0001
• Has difficulty changing attention	45,85	90,48	
**Weakness in Power Movement**			
• Seem to have weak muscles (not trained)	62,60	73,48	0,005
• Easily tired especially when standing / using the limbs	60,63	75,49	0,0001
• Has a weak hand grip	58,65	77,49	0,0001
• No ability to lift heavy objects	65,05	70,99	0,031
• Need a backrest to help him / her	63,58	72,49	0,010
• Weak resistance / easy to feel fatigue	65,56	70,48	0,123
**Visual/Auditory Sensitivity **			
• Respond negatively to loud sounds that are suddenly heard	51,76	84,48	0,0001
• Often closing the ears to protect the ear from noise	59,19	76,94	0,0001
• Easily glare or uncomfortable with sunlight or bright lights	67,54	68,46	0,773
• Observe the movements of everyone around the room	56,24	79,94	0,0001
• Often rub or squint to protect eyes from light	65,13	70,92	0,221

The development of the somatosensory system in early infancy is hypothesized to be foundational for social and communication skills later in life (13). However, neurodevelopmental abnormalities in the brain may have a targeted influence on symptoms associated with ASD occurred in autism (14). Certain neurological development disorders in the brain cause many problems in processing tactile sensory input, causing sensations from the environment that are normally recorded and interpreted in the brain or central nervous system to be distracted: unable to filter inputs, often failing to process important information and prone to stress and anxiety (5,10). The neurobiological mechanisms against the incidence of abnormality of tactile system and symptoms of ASD are still not known clearly and definitely. These abnormalities may be exacerbated due to the dysfunction in the excitation/inhibition balance of the central nervous system of those with ASD (15). 

**In conclusion, **autistic children are more sensitive to touch, pain, and all categories of SSP than children who are not autistic. Basic understanding of children with ASD will help the parent in handling in daily life. This study finding could be used to recover motoric of children with ASD. The parents have main role in handling ASD children. Further research is necessary to explore more sample and comprehensive study by considering the social background of family. The information of relation between social background and ASD children condition could resolve this issue.
